# Supplementary material for: Temporin L and aurein 2.5 have identical conformations but subtly distinct membrane and antibacterial activities
Source: Sci Rep. 2019 Jul 29;9:10934. doi: 10.1038/s41598-019-47327-w (PMC6662694; doi:10.1038/s41598-019-47327-w)
Supplement: Supplementary file 1 — Supplementary Figures [file 41598_2019_47327_MOESM1_ESM.pdf]

Temporin L and aurein 2.5 have identical conformations but subtly distinct membrane and antibacterial activities

Giorgia Manzo[1], Philip M. Ferguson[1], Charlotte Hind[2], Melanie Clifford[2], V. Benjamin Gustilo[1], Hind Ali[1], Sukhvinder S. Bansal[1], Tam T. Bui[3], Alex F. Drake[3], R. Andrew Atkinson[3], J. Mark Sutton[2], Christian D. Lorenz[4], David A. Phoenix[5], A. James Mason[1*]

[1]Institute of Pharmaceutical Science, School of Cancer & Pharmaceutical Science, King's College London, Franklin-Wilkins Building, 150 Stamford Street, London, SE1 9NH, United Kingdom

[2]Technology Development Group, National Infection Service, Public Health England, Salisbury, UK

[3]Centre for Biomolecular Spectroscopy and Randall Division of Cell and Molecular Biophysics, King's College London, New Hunt's House, London SE1 1UL, United Kingdom

[4]Department of Physics, King's College London, London WC2R 2LS, United Kingdom

[5]School of Applied Science, London South Bank University, 103 Borough Road, London SE1 0AA, United Kingdom

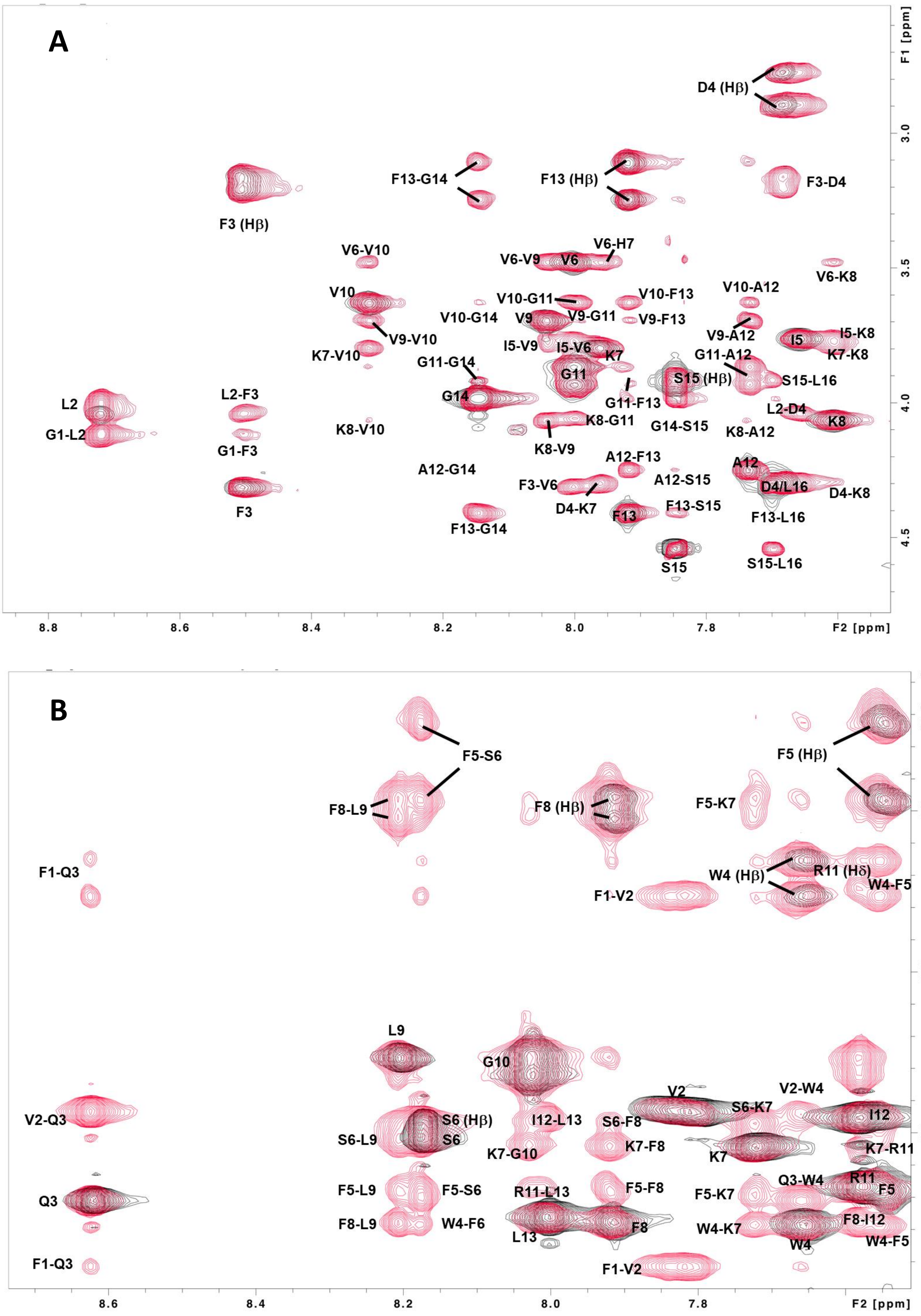

Figure S1. 2D NMR spectra obtained at 500 MHz for ^{1}H and used for structure determination of the two AMPs. Overlays of the fingerprint region of 2D TOCSY (black) and 2D NOESY (red) spectra are shown for aurein 2.5 (A) and temporin L (B) with assignments. In each case the samples were 2 mM peptide in 100 mM SDS-d_{25}.

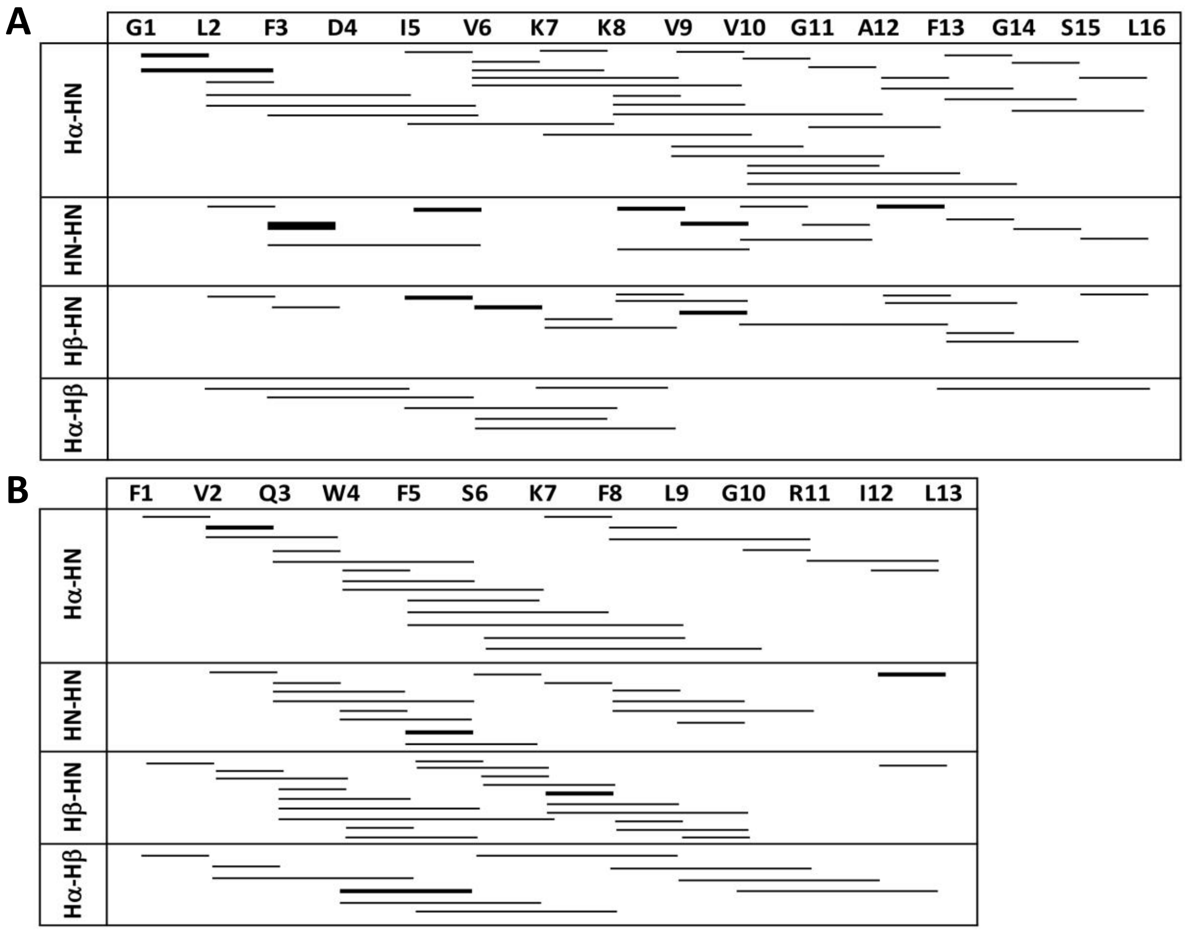

Figure S2. NOE table for aurein 2.5 (A) and temporin L (B) in SDS. The table shows sequential NOE connectivities between H_{α}-H_N, H_{β}-H_N and H_N-H_N where the weight of each line corresponds to the cross-peak intensity shown in the ^{1}H-NOESY spectrum. A thin line represents a weak range connection, a medium-width line represents a medium-range connection and a thick line represents a strong range connection.

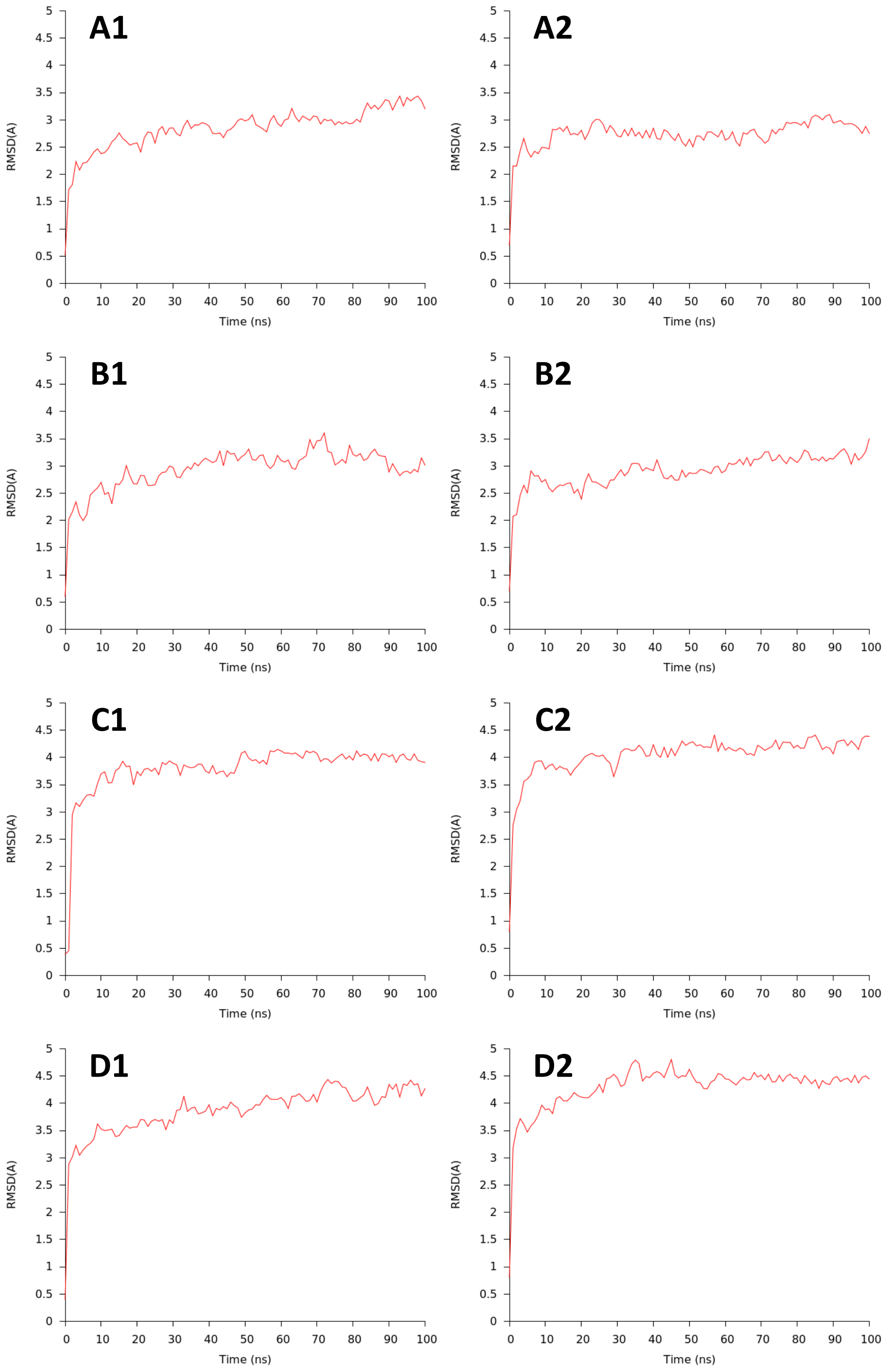

Figure S3. Change in average AMP root mean squared deviation (RMSD) over the 100 ns of each of the eight simulations; aurein 2.5 (A/B) or temporin L (C/D) binding to POPE/POPG (A/C) or POPG (B/D). The RMSD is calculated by taking the starting (NMR) structure, aligning this to each of the eight peptides and comparing over the course of the trajectory. The average is then plotted. The most substantial structural changes are complete within a few ns as the peptide binds to the membrane and the RMSD plateaus before the end of each 100 ns simulation.

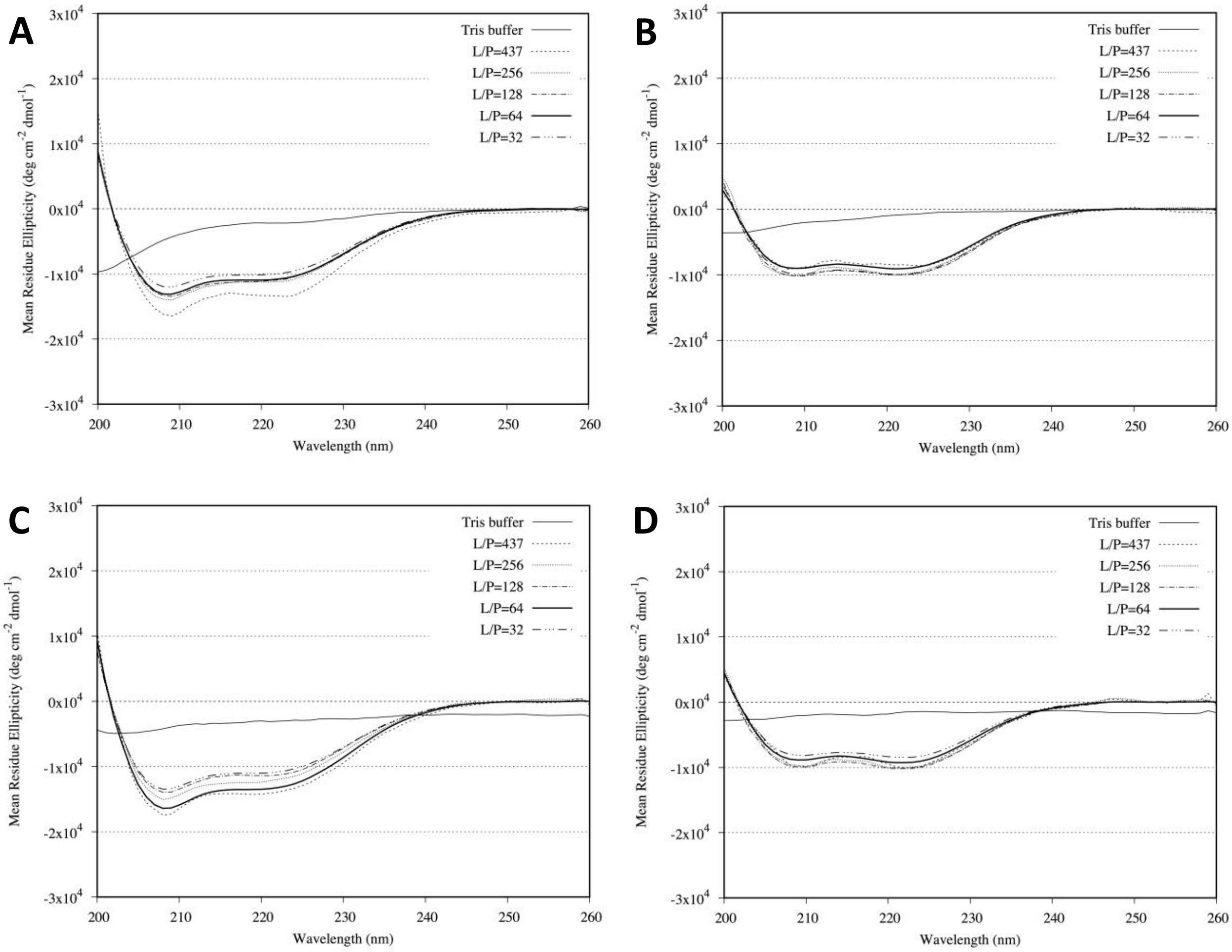

Figure S4. Dose dependent variations in secondary structure in model membranes. Far-UV CD spectra of aurein 2.5 (A/C), temporin L (B/D) were acquired in the presence of 5 mM POPE/POPG (75/25) (A/B) or POPG (C/D) SUVs, 5 mM Tris-amine buffer, pH 7.00 and 100 mM NaCl.

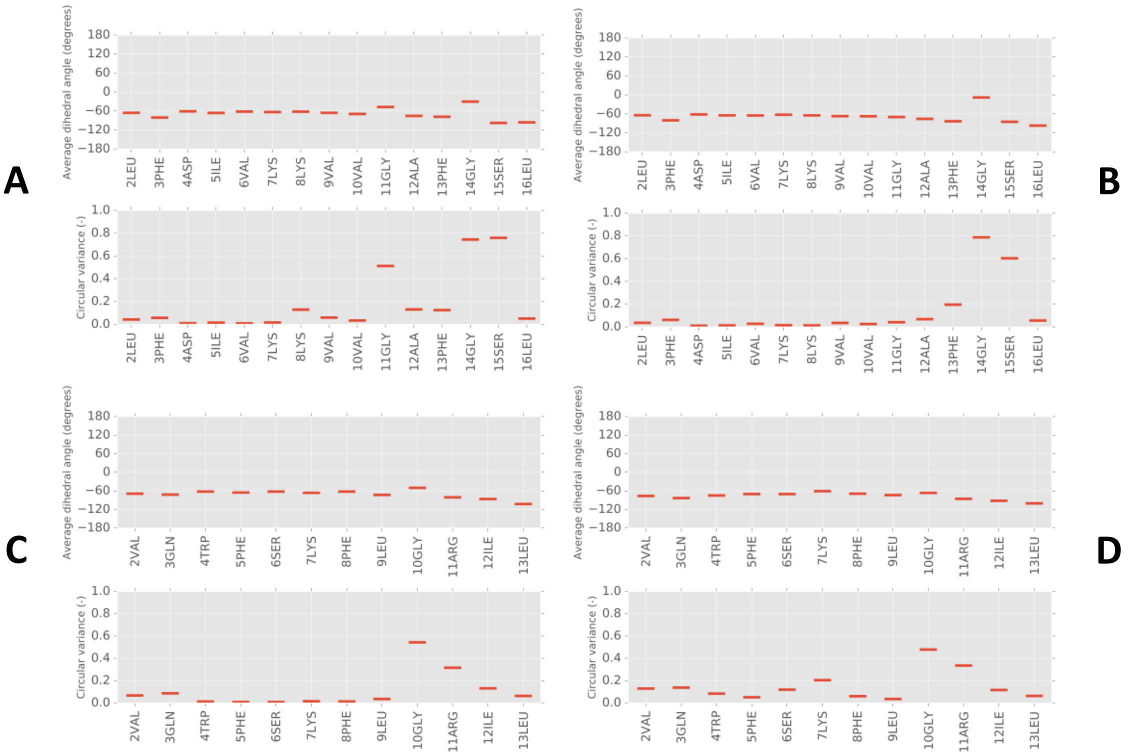

Figure S5. Secondary structure analysis of aurein 2.5 and temporin L peptides from MD simulations. Mean dihedral angles (phi) (upper plot) and their circular variance (lower plot) are shown for each residue to enable identification of regiospecific changes in conformation and/or flexibility. Angles were averaged over 100 ns of simulation and eight peptides in aurein 2.5 (A/B) and temporin L (C/D) peptides when binding to POPE/POPG (A/C) or POPG (B/D) membranes.

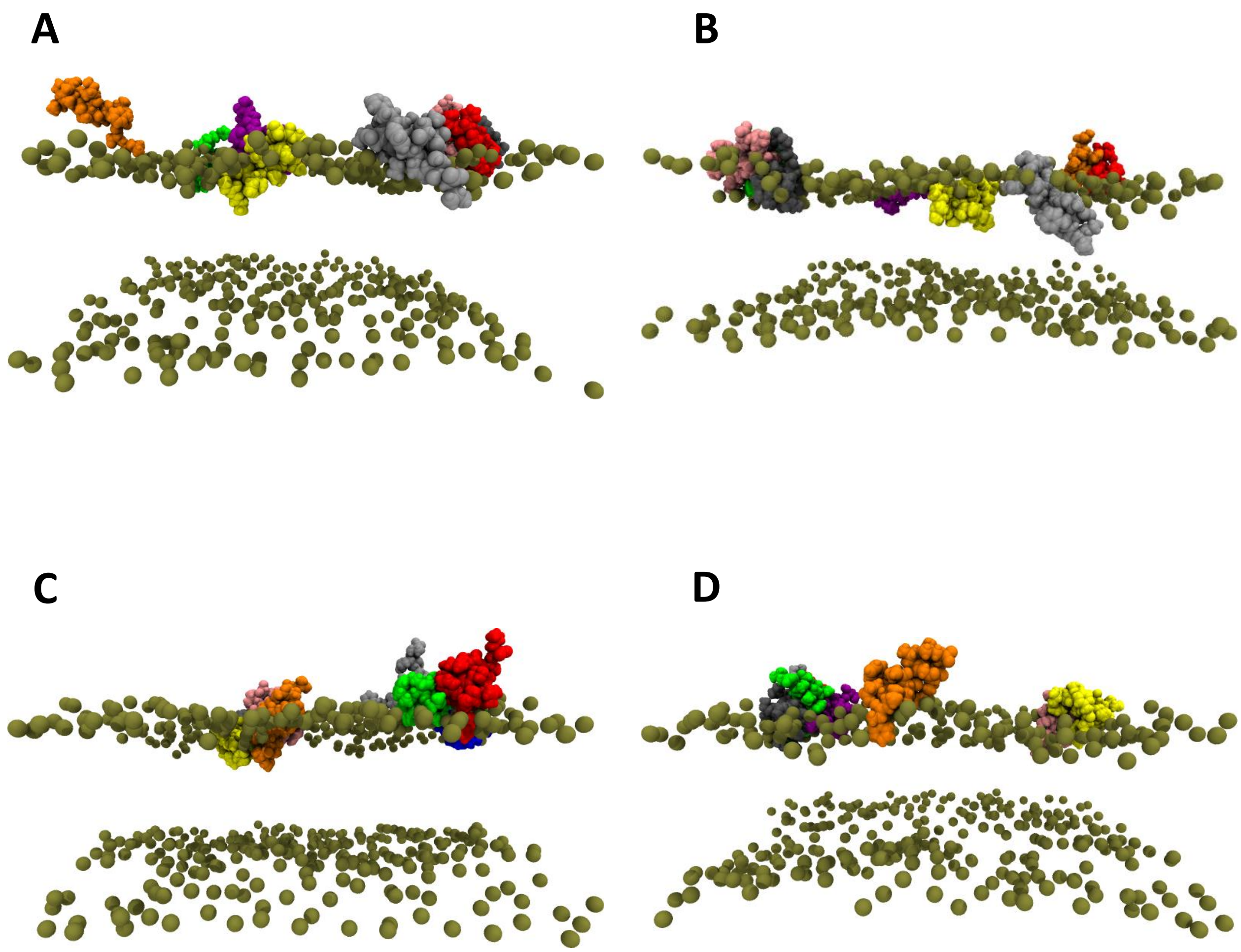

Figure S6. Interaction of aurein 2.5 and temporin L with bilayers. The depth of peptides insertion is shown in the side view snapshot for POPE/POPG (A/C) or POPG (B/D for aurein 2.5 (A/B) and temporin L (C/D).

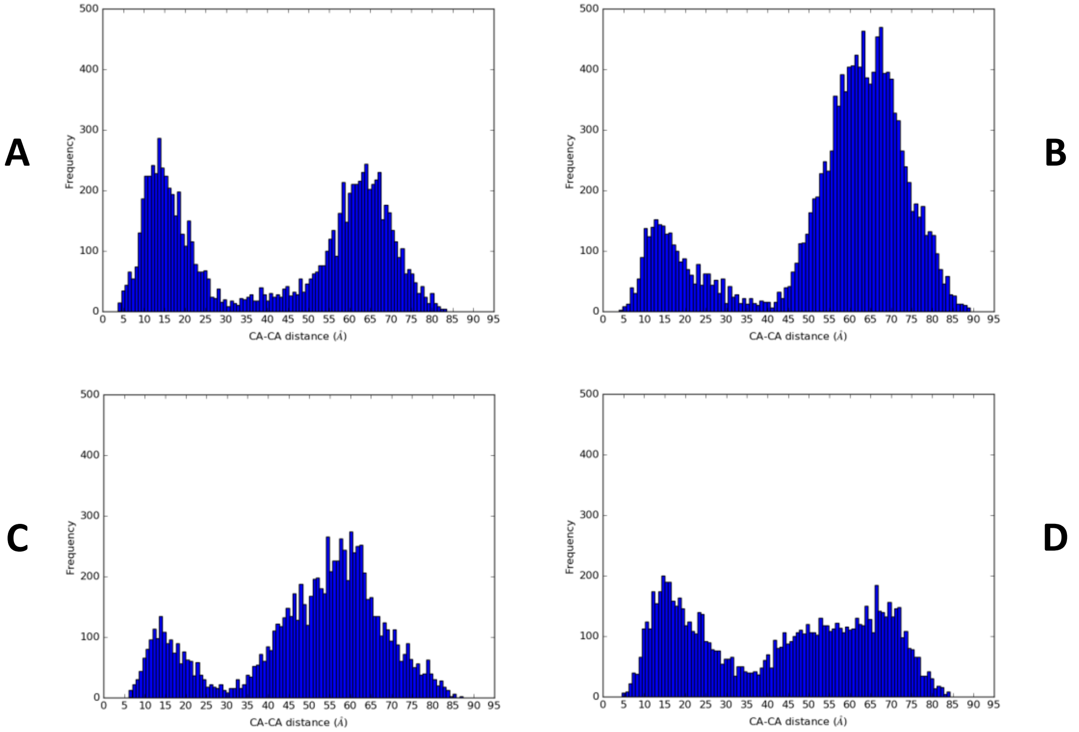

Figure S7. Aurein 2.5 and temporin L peptide aggregation during 100 ns MD simulation. The probability of aurein 2.5 (A/B) and temporin L (C/D) peptide aggregation is reported as frequency histogram of the Cα-Cα distances, respectively for peptides in POPE/POPG (A/C) or POPG (B/D) membranes.

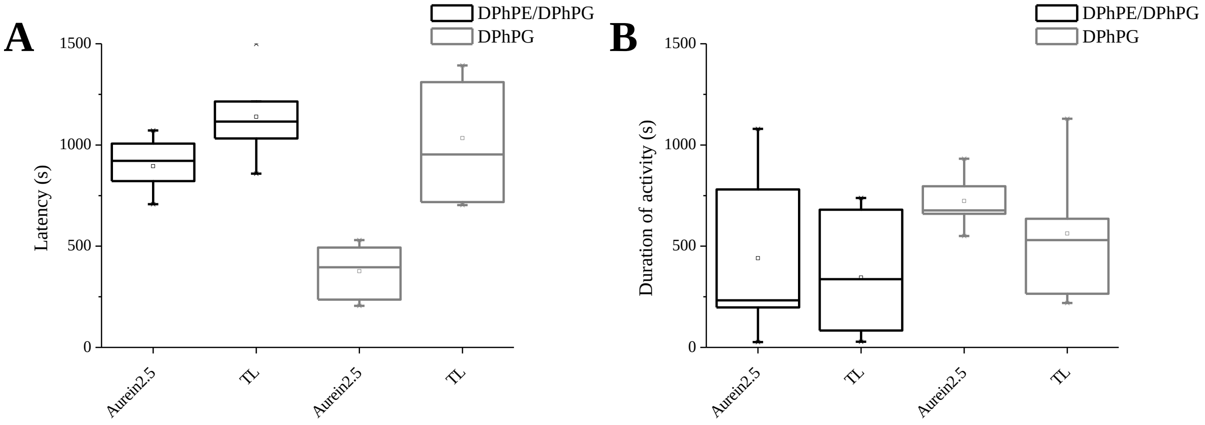

Figure S8. Latency and duration of membrane activity. The average time elapsed between peptide addition and the first appearance of membrane activity (A) and the average duration of activity before the membrane breaks (B) is shown for each peptide in DPhPE/DPhPG or DPhPG membranes.

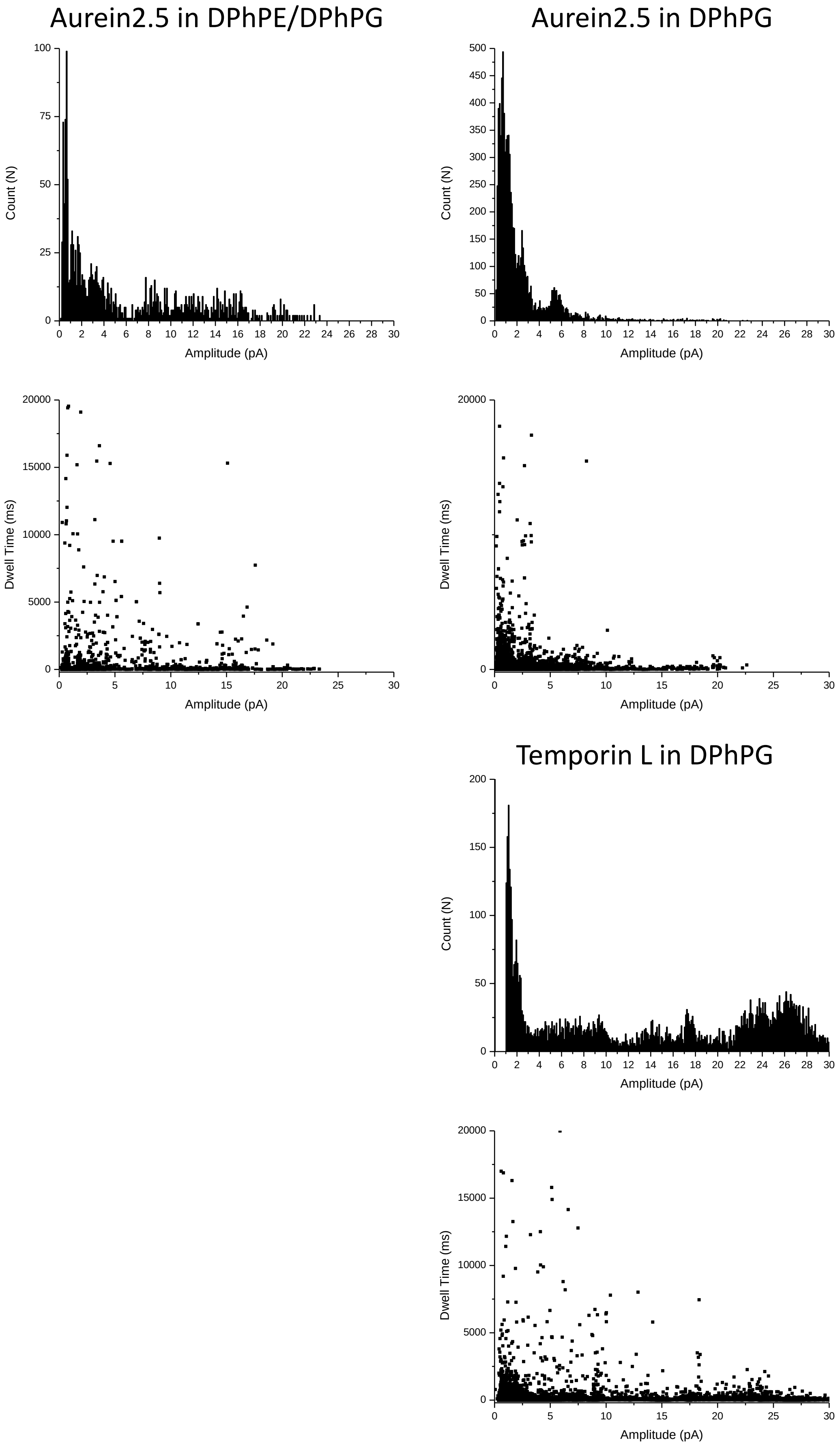

Figure S9. Channel-like events induced by aurein 2.5 or temporin L. For current traces where channel-like activity was detected this is described as either a histogram, to reveal levels with channels of a defined amplitude, or dwell time is plotted as a function of amplitude. Channel-like activity was not detected for temporin L in DPhPE/DPhPG.
